# Brain death in a neurologic intensive care unit in turkey

**DOI:** 10.1186/2197-425X-3-S1-A987

**Published:** 2015-10-01

**Authors:** Z Kurtpınar, T Akbaş, S Şenadım, E Çoban, V Özkubat, SK Bayrak, HA Güllü, A Soysal, D Ataklı

**Affiliations:** Bakirkoy Research and Training Hospital for Psychiatry, Neurology and Neurosurgery, Istanbul, Turkey; Bakirkoy Research and Training Hospital for Psychiatry, Neurology and Neurosurgery, Intensive Care Unit, Istanbul, Turkey; School of Medicine, Internal Medicine and Intensive Care Unit, Düzce University, Duzce, Turkey

## Introduction

The diagnosis of brain death (BD) is important in order to decrease the burden of futile care and procure vital and viable organs for donation. The aim of this study was to find clinical features of patients, diagnostic tools used, attitude to BD and donation rate in a teaching hospital for neurologic diseases in Istanbul.

## Methods

All consecutive BD patients between April 2014-April 2015 were included in this retrospective study. Data were abstracted from medical records. Statistical analyses were performed using SPSS software. The results were expressed as percentages for categorical data and median with interquartile range for continuous variables.

## Results

Twenty-three patients [10F/13M, age: 63 (54-75)] had BD diagnosis. Neurologic etiologies were ischemic stroke (52%), intracranial bleeding (39%), hypoxic encephalopathy (4.5%) and transverse sinus thrombosis (4.5%). The Glasgow Coma Scales (GCS) were 9 (5-11) at hospital and 7 (5-9) at ICU admission. The clinical sign encouraging the clinicians to think BD were brain stem areflexia (39%), decreased GCS (34.5%), absence of spontaneous breathing (17.5%) and polyuria (9%). BD was suspected first by ICU physicians in 56.5%. BD diagnosis was delayed for > 12 hours in 11 patients because of being sure about BD (46%), legal observation time (27%), sedation (18%) and hemodynamic instability (9%). Fifteen patients had BD diagnosis with apnea and brainstem testing. The remaining 8 cases had confirmatory diagnostic evaluations of BT angiography (6) and EEG (2) due to inability of performing apnea testing (5), clinical suspicion (2) and uncomfortable feeling in giving the decision (1). Apnea testing was reported before neurologic examination in 26%. No complication was documented during apnea testing. Diabetes insipidus was developed in 78% and desmopressin was given to 39% of polyuric patients. Vasopressors were ordered to 95.5% [Noradrenaline (68%), dopamine (18%) and both drugs (14%)] and methylprednisolone [60 (36-60) mg/day] to 57%. Levothyroxine [300 (112-400) mcg] was ordered only one time to 39% during the BD diagnosis period. Three (13%) patients were allowed to be donors by the relatives. The therapy was withdrawn in 55% and held on the present support until cardiac arrest in 35% of the nondonors. No information was found for 10% of the nondonors. The time span from BD confirmation to cardiac arrest developed was 120 (60-636) minutes for the nondonors.

## Conclusions

The main clinical signs encouraging the clinicians to identify brain death are brainstem areflexia and decrease in GCS. Apnea and brain stem testing are the major diagnostic approach. Diabetes insipidus and hypotension are commonly encountered situations in these patients.
